# Intermediate-dose cytarabine is an effective therapy for adults with non-Langerhans cell histiocytosis

**DOI:** 10.1186/s13023-022-02193-0

**Published:** 2022-02-07

**Authors:** Ting Liu, Hua-cong Cai, Hao Cai, Miao Chen, Wei Zhang, Jian Li, Dao-bin Zhou, Xin-xin Cao

**Affiliations:** 1 Department of Hematology, Peking Union Medical Hospital, Dongcheng District, Chinese Academy of Medical Sciences and Peking Union Medical College, 1 Shuai Fu Yuan Hu Tong, Beijing, 100730 People’s Republic of China; 2 State Key Laboratory of Complex Severe and Rare Diseases, Peking Union Medical College Hospital, Chinese Academy of Medical Sciences and Peking Union Medical College, Beijing, 100730 People’s Republic of China

**Keywords:** Non-Langerhans cell histiocytosis, Cytarabine, Efficacy

## Abstract

**Background:**

Non-Langerhans cell histiocytosis, including Erdheim–Chester disease (ECD), Rosai–Dorfman disease (RDD), indeterminate cell histiocytosis (ICH), and unclassified histiocytosis, is a rare disorder lacking a standard treatment strategy. We report our experience using intermediate-dose cytarabine as the first or subsequent therapy in non-Langerhans cell histiocytosis.

**Results:**

Nine ECD patients, 5 RDD patients, 1 ICH patient and 1 unclassified histiocytosis patient were enrolled. Intermediate-dose cytarabine therapy was administered as 0.5–1.0 g/m^2^ of intravenous cytarabine every 12 h for 3 days every 5 weeks. The median age at cytarabine initiation was 47.5 years (range 18–70 years). The median number of cycles of cytarabine administered was 5.5 (range 2–6). The overall response rate (ORR) was 87.5% in the overall cohort, including 12.5% with complete response and 75.0% with partial response. One patient experienced disease recurrence 19 months after cytarabine therapy. The median follow-up duration for the entire cohort was 15.5 months (range 6–68 months). The estimated 2-year progression-free survival and overall survival rates were 85.6% and 92.3%, respectively. The most common toxicity was haematological adverse events, including grade 4 neutropenia and grade 3–4 thrombocytopenia. No treatment-related deaths occurred.

**Conclusions:**

Intermediate-dose cytarabine is an efficient treatment option for non-Langerhans cell histiocytosis patients, especially for those with CNS involvement.

## Introduction

Histiocytosis is a rare disorder characterized by the accumulation of macrophages, dendritic cells, or monocyte-derived cells in various tissues and organs [1]. According to the 2016 revised histiocytosis classification [1], histiocytosis are classified into five categories: L Group: Langerhans cell histiocytosis (LCH), Erdheim-Chester disease (ECD), and indeterminate cell histiocytosis(ICH); C Group: non-LCH histiocytosis involving skin or mucosa and comprising both xanthogranuloma and non-xanthogranuloma subtypes; M Group: primary and secondary malignant histiocytosis; R Group: Rosai-Dorfman disease and other noncutaneous, non-LCH histiocytosis; H Group: hemophagocytic lymphohistiocytosis (HLH). Their heterogeneity and rarity pose great challenges to the establishment of standard treatment strategies.

The discovery of the BRAF^V600E^ mutation in approximately 50% of patients with LCH [2] and ECD [3] provided the first molecular therapeutic target in histiocytosis. BRAF inhibition is highly efficacious and has markedly altered the natural history of these disorders [4]. For patients who lack BRAF^V600^ mutations but carry other MAPK-ERK pathway mutations, treatment with MEK inhibitors has shown clinical efficacy [5]. However, recurrent driving mutations of the MAPK/ERK pathway are not universal in non-Langerhans cell histiocytosis [6], and recent biological and molecular advances in ECD have not been matched in other non-Langerhans disorders. For non-Langerhans cell histiocytosis types excluding ECD, the efficacy of targeted therapy has only been reported in isolated case reports.

Cytarabine is an efficient cytotoxic drug that plays an important role in the treatment of haematological neoplasms, including histiocytic neoplasms. We previously reported remarkable responses to intermediate-dose cytarabine in 3 patients with ECD [7, 8] and 2 patients with RDD [9] with central nervous system (CNS) involvement.

Here, we conduct a retrospective review of the use of intermediate-dose cytarabine in adults with non-Langerhans cell histiocytosis, including ECD, RDD, ICH and unclassified histiocytosis, to analyse the efficacy and safety of cytarabine in these patients.

## Methods

### Patients

A retrospective review was conducted among patients who were diagnosed with non-Langerhans cell histiocytosis (ECD, RDD, ICH and unclassified histiocytosis) and had received intermediate-dose cytarabine for at least 2 cycles at Peking Union Medical College Hospital between October 2013 and August 2021. The diagnosis of non-Langerhans cell histiocytosis was based on typical clinical presentation, radiologic presentation, and histologic findings that were reviewed independently by two pathologists. Informed consent was obtained from all patients, and the protocol was approved by the Peking Union Medical College Hospital Ethics Committee. The present study was performed in accordance with the ethical standards of the 1964 Declaration of Helsinki and its later amendments.

### Clinical, imaging, and genetic data

Clinical data were collected regarding age, sex, lesion location, physical examination, laboratory data, treatment, and survival. Imaging data were collected from FDG-PET; computed tomography (CT) of the entire aorta, chest, abdomen and pelvis; and magnetic resonance imaging (MRI) of the brain and heart. DNA extracted from formalin-fixed and paraffin-embedded preserved lesion biopsy samples of the enrolled patients was obtained and subjected to next-generation sequencing of 183 genes as previously described [10]. The presence of the BRAF^V600E^ mutation was detected by polymerase chain reaction (PCR) or immunohistochemistry in some cases as previously described [11].

### Treatment, response and toxicity criteria

Intermediate-dose cytarabine therapy was defined as the administration of 0.5–1.0 g/m^2^ of intravenous cytarabine every 12 h for 3 days every 5 weeks for 4–6 cycles in total. All patients were followed up every 3–6 months. Response assessment was primarily performed using the PET Response Criteria in Solid Tumors (PERCIST) [12], and the patients were then classified as having complete metabolic response (CMR, complete resolution of pathologic FDG uptake), partial metabolic response (PMR, reduction of a minimum of 30% in activity of the target lesions), stable metabolic disease (SMD, not complete or partial metabolic response), or progressive metabolic disease (PMD, increase of a minimum of 30% in the activity of the target lesions or the presentation of a new lesion). Patients unable to undergo FDG-PET underwent response assessment using CT or MRI according to the Response Evaluation Criteria in Solid Tumors (RECIST; version 1.1) [13]. Responses were categorized as follows: complete response (CR): disappearance of all target lesions; partial response (PR): at least a 30% decrease in the sum of the diameters of the target lesions; progressive disease (PD): at least a 20% increase in the sum of the diameters of the target lesions; and stable disease (SD): neither sufficient shrinkage to qualify for PR nor sufficient increase to qualify for PD. Chemotherapy-related toxicities were assessed using the Common Terminology Criteria for Adverse Events (CTCAE) version 4.03 (National Cancer Institute, Bethesda, MD).

### Statistical analysis

The follow-up was conducted up to January 6, 2022. Overall survival (OS) was defined as the time from the date of cytarabine treatment to the date of death or the last follow-up. Progression-free survival (PFS) was calculated from the date of cytarabine treatment until the date of disease progression, relapse, or death from any cause. We performed all statistical analyses using SPSS version 21 (IBM Corp., Armonk, NY, USA). Kaplan–Meier analysis was used for survival analysis, with the survival curves compared using the log-rank test.

## Results

### Patients

A total of 16 patients (10 males and 6 females) met the inclusion criteria. The patients were diagnosed with ECD (n = 9), RDD (n = 5), ICH (n = 1) and unclassified histiocytosis (n = 1, Table 1). The median age at cytarabine initiation was 47.5 years (range 18–70 years). Thirteen (81.25%) patients had multisystem disease, and the most commonly involved organs were the CNS (68.8%), bones (68.8%), retroperitoneum (including the kidneys, 31.3%), orbit (25.0%), vasculature(25.0%), thyroid (18.8%), and pericardium (18.8%).

**Table 1:** Patient descriptions

Patient #	Diagnosis	Age at cytarabine initiation (years)	Gender	Sites of disease	Gene mutations	Previous therapies	Total number of cytarabine cycles	Response	Progression or disease recurrence? (time from start of therapy to relapse)	Follow-up duration (months)	Maintenance or recurrent treatment
1	ECD	45	F	CNS, bones, pituitary	None-detected	None	4	PR	No	48	Interferon-α
2	ECD	24	F	CNS, bones, obit, lung, retroperitoneum	BRAF ^V600E^	None	4	PR	No	39	Interferon-α
3	ECD	56	M	bones, vasculature, orbit, lung, nasal sinus, retroperitoneum	BRAF ^V600E^	Interferon-α	6	PR	No	13	Interferon-α
4	ECD	36	F	bones, vasculature, orbit, thyroid, pericardium, retroperitoneum	None-detected	Interferon-α	4	PD	Yes (5 months)	12	Sirolimus/prednisone
5	ECD	50	M	CNS, bones, pituitary, thyroid, vasculature, nerve root	NA	None	6	PR	No	17	Interferon-α
6	ECD	51	F	CNS, bones	BRAF ^V600E^	None	6	PR	Yes (19 months)	25	Interferon-α → vemurafenib
7	ECD	53	F	Bones, heart, pericardium	BRAF ^V600E^	Interferon-α	2	PD (died)	Yes (6 months)	6	NA
8	ECD	51	F	CNS, bones, skin, thyroid, retroperitoneum, pancreas	None-detected	None	4	PR	No	9	Interferon-α
9	ECD	52	M	CNS, Bone, pericardium, orbit, retroperitoneum, vasculature, lung	None-detected	Interferon-α	5	PR	No	68	Interferon-α
10	ICH	22	M	Bones, CNS (Spinal cord)	NA	None	6	PR	No	14	NA
11	RDD	34	M	CNS	None-detected	None	6	PR	No	23	Lenalidomide/dexamethasone
12	RDD	37	M	CNS, dura	NA	Surgery	6	CR	No	12	NA
13	RDD	70	M	Bones, orbit, nasal sinus, glottis	BRAF ^R188G^ MAP2K1^D147G1^	Methylprednisolone	4	PR	No	8	Lenalidomide/dexamethasone
14	RDD	18	M	CNS, pituitary	BRAF ^V600E^ wild type	None	6	PR	No	61	Interferon-α
15	RDD	50	M	CNS, dura	NA	Methylprednisolone/Rituximab	6	PR	No	60	Interferon-α
16	Unclassified histiocytosis	22	M	Bones, skin, gingiva	NA	COP, CHOP, lenalidomide	3	PR	No	6	NA

COP: Cyclophosphamide/vincristine/prednisone; CHOP: Cyclophosphamide/epirubicin/vincristine/predisone

We performed next-generation sequencing on 6 patients with ECD and 2 patients with RDD. No pathogenic mutations were detected in 3 patients with ECD, and the remaining 3 patients had the BRAF^V600E^ mutation. Mutations of BRAF^R188G^ at a variant-allele frequency of 4.3% and MAP2K1^D147G1^ at a variant-allele frequency of 5.4% were detected in one RDD patient, while no pathogenic mutations were detected in the other RDD patient. Since the RDD patient carried BRAF^R188G^ rather than BRAF^V600E.^ and lacked typical clinical presentation of ECD, we don’t think he is not a mixed histiocytosis (ECD/RDD). The BRAF^V600E^ status was detected by PCR in 3 patients, and one patient with ECD had the BRAF^V600E^ mutation, while another two patients were BRAF^V600E^ wild type (1 ECD and 1RDD). Two patients with no detectable gene mutation performed immunohistochemistry for BRAF^V600E^, and the stain were negative.

### Treatment and response

Cytarabine was administered as frontline systemic therapy in 8 (50.0%) patients and as subsequent-line treatment in 8 (50.0%) patients. Of the 8 patients who received cytarabine in the subsequent line, prior therapies included interferon-α (n = 4), polychemotherapy (n = 2), corticosteroids (n = 1), and surgery (n = 1, Table 1). The median number of cycles of cytarabine administered was 5.5 (range 2–6). Response assessment was conducted using FDG-PET in 10 (62.5%) patients, and the response rates were as follows: CMR, 10.0% (n = 1); PMR, 80.0% (n = 8); and PMD, 10.0% (n = 1). The remaining 6 patients were assessed by CT or MRI; 5 patients achieved PR (83.3%), and 1 patient had PD and died. Therefore, the overall response rate (ORR) was 87.5% (n = 14) in the overall cohort. Responses were seen in various disease sites: CNS (90.90%), naval sinus (100%), orbit (50%), thyroid(33.3%), vasculature (25.0%), bones (27.3%).

### Survival and toxicity

**Fig. 1:**
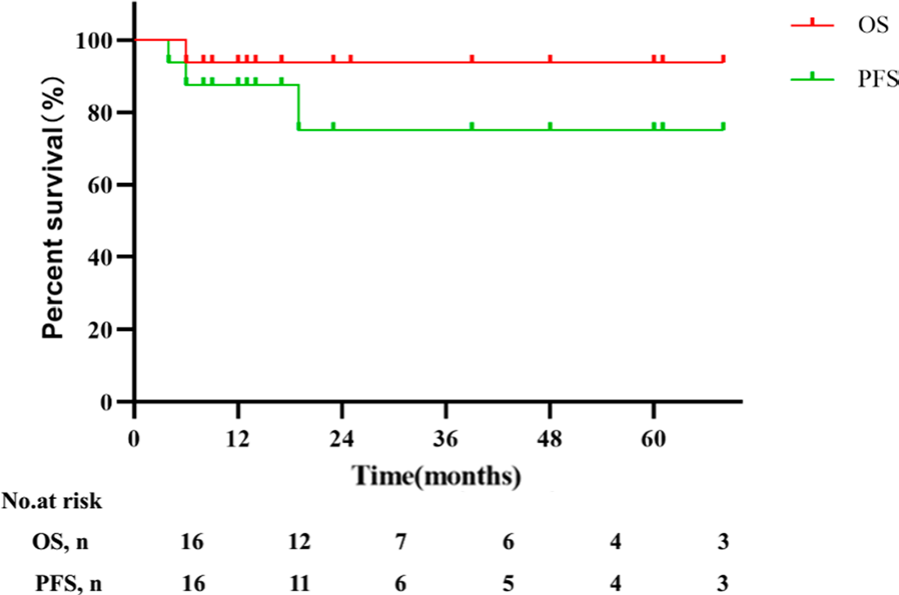
Progression-free survival (PFS) and overall survival (OS) for the whole cohort (n = 16)

The median follow-up duration for the entire cohort was 15.5 months (range 6-68 months). After cytarabine therapy, 9 patients received interferon-α (IFN-α), and 2 patients received lenalidomide plus dexamethasone for maintenance. One patient with ECD experienced recurrence during the maintenance treatment of IFN-α (19 months after cytarabine therapy) and was switched to vemurafenib therapy. The patient who did not respond to cytarabine therapy was then treated with sirolimus and prednisone, and the patient's condition was stable up to the last follow-up. The estimated 2-year PFS and OS rates were 75.0% and 93.5%, respectively (Fig. 1).

The most common toxicity was haematological adverse events, and blood count abnormalities were retrospectively graded according to the CTCAE version 4.03. All patients experienced grade 4 neutropenia, and 9 patients experienced neutropenic fever. Eight patients developed grade 3–4 thrombocytopenia, but no severe bleeding events occurred. Drug fever induced by cytarabine occurred in 2 patients, and the temperature returned to normal after antipyretic treatment. None of the patients received anti-infective prophylaxis for or developed Pneumocystis jiroveci pneumonia. Treatment delays or dose-reductions related to adverse effects did not occur and no treatment-related deaths occurred.

## Discussion

Non-Langerhans cell histiocytosis is a rare disorder lacking a standard treatment strategy. Due to the discovery of activating and targetable MAPK-ERK pathway mutations in the vast majority of patients with ECD, the therapeutic landscape of ECD has changed drastically over the last decade [3, 4]. BRAF inhibitors, such as vemurafenib or dabrafenib, are recommended as first-line therapy for patients with multisystem BRAF-V600-mutant ECD who have life-threatening cardiac or neurologic involvement, leading to response rate of nearly 100%; for patients without BRAF-V600 mutation, NGS is suggested to evaluate other MAPK-ERK pathway alterations that can be treated with a MEK inhibitor [14]. However, BRAF and MEK inhibitors are costly (far beyond what most patients in China can afford) and are not covered by health insurance in China.

Treatment is reserved for symptomatic disease or multisystemic involvement for patients with RDD. The conventional systemic therapies for RDD include steroids, chemotherapy, sirolimus, and immunomodulatory therapy, such as thalidomide and lenalidomide [15]; however, the efficacy of steroids or other systemic therapies for RDD is variable. MEK inhibitors such as cobimetinib used in patients with MAPK-ERK pathway alterations seem promising [5], but have limited experience. Data regarding the efficacy and safety of systemic treatments for other non-Langerhans cell histiocytic disorders, such as ICH and JXG, are especially lacking. Overall, the management of patients with non-Langerhans cell histiocytosis is challenging. Systemic nontargeted therapy for patients with non-Langerhans cell histiocytosis deserves exploration.

Our previous data suggested that CNS involvement was a poor prognostic factor for ECD patients with INF-α [16]. Therefore, we attempted to explore treatments other than IFN-a for non-Langerhans cell histiocytosis, especially drugs that can penetrate the blood–brain barrier. Cytarabine easily penetrates the blood–brain barrier and has shown promising therapeutic prospects in non-Langerhans cell histiocytic disorders [8, 9].

The patients enrolled in the present study had multisystem involvement with or without previous therapy. Most relapsed patients in this study accepted standard first-line treatments, as reported in the literature [14, 15]. Although some patients in our study carried the BRAF^V600E^ mutation, they could not afford BRAF inhibitors as first-line treatment. We found that cytarabine has favourable clinical efficacy in non-Langerhans cell histiocytosis patients with multisystem involvement regardless of its use as frontline therapy or subsequent-line treatment. The overall clinical response rate was 87.5%, with estimated 2-year PFS and OS rates were 75.0% and 93.5%, respectively. These outcomes are much better than those of other non-targeted therapies for ECD (ORR of IFN-a [16, 17], cladribine [18] and anakinra [19] were 67–80%, 52% and 50% respectively) and RDD (ORR of corticosteroids and cladribine were 56% and 67% respectively [20]). Impressive responses were seen in patients with central nervous system involvement.

In terms of regimen toxicity, grade 4 neutropenia was noted in all patients, and grade 3–4 thrombocytopenia occurred in 53.3% of patients during treatment. Nevertheless, no treatment-related deaths occurred. It is critical to monitor routine blood tests regularly and use granulocyte colony-stimulating factor or transfuse platelets according to routine blood results.

This study’s limitations include the small number of patients, the retrospective nature of the analysis, the lack of long-term follow-up for outcomes and toxicity, and incomplete description of toxicity including organ toxicity. Nevertheless, large-scale cohort studies or prospective clinical trials are of great difficulty due to the rarity of the disease. We will extend the follow-up duration to monitor the long-term outcomes and toxicity.

## Conclusion

In conclusion, intermediate-dose cytarabine is an efficient and safe treatment option for non-Langerhans cell histiocytosis, especially for patients with CNS involvement.

## Data Availability

The datasets used and/or analysed during the current study are available from the corresponding author on reasonable request.

## Abbreviations

CNS: Central nervous system
CMR: Complete metabolic response
CR: Complete response
CT: Computed tomography
CTCAE: Common Terminology Criteria for Adverse Events
ECD: Erdheim–Chester disease
FDG-PET: 18Ffluorodeoxyglucose positron emission tomography
ICH: Indeterminate cell histiocytosis
IFN-α: Interferon-α
LCH: Langerhans cell histiocytosis
JXG: Juvenile xanthogranuloma
MRI: Magnetic resonance imaging
PCR: Polymerase chain reaction
PERCIST: PET Response Criteria in Solid Tumors
PD: Progressive disease
PFS: Progression-free survival
PR: Partial response
PMR: Partial metabolic response
PMD: Progressive metabolic disease
RECIST: Response Evaluation Criteria in Solid Tumors
RDD: Rosai–Dorfman disease
ORR: Overall response rate
OS: Overall survival
SD: Stable disease
SMD: Stable metabolic disease
